# Impact of gluten-free diet (GFD) on some of cardiovascular risk factors: a systematic review and meta-analysis

**DOI:** 10.1017/jns.2024.39

**Published:** 2024-09-18

**Authors:** Pejman Rohani, Elma Izze da Silva Magalhães, Roya Imanifard, Maryam Jarahzadeh, Fateme Ziamanesh, Somaye Fatahi, Hajar Ghorbani Jalalieh, Mohammad Hassan Sohouli

**Affiliations:** 1 Pediatric Gastroenterology and Hepatology Research Center, Pediatrics Centre of Excellence, Children’s Medical Center, Tehran University of Medical Sciences, Tehran, Iran; 2 Postgraduate Programme in Collective Health, Federal University of Maranhão, São Luís, MA, Brazil; 3 Iran University of Medical Sciences, Tehran, Iran; 4 Department of Clinical Nutrition and Dietetics, Faculty of Nutrition and Food Technology, Shahid Beheshti University of Medical Sciences, Tehran, Iran; 5 Student Research Committee, Department of Clinical Nutrition and Dietetics, Faculty of Nutrition and Food Technology, Shahid Beheshti University of Medical Sciences, Tehran, Iran

**Keywords:** Cardiovascular, Gluten-free diet, Glycaemic, Inflammation, Lipid profile, Meta-analysis

## Abstract

A gluten-free diet (GFD) may have a stronger potential impact on reducing cardiovascular (CV) risk factors, according to research evidence. We investigated the impact of GFD on CV risk variables by doing a systematic review and meta-analysis for this reason. We conducted a thorough database search starting on January 1, 2000, and ending on July 12, 2022. We used random-effects models to pool the data. Totally 19 articles met the eligible criteria and were included. Pooled findings indicated that intervention with GFD has a significantly beneficial effect on high-density lipoprotein (HDL) (WMD: 4.80 mg/dl, 95% CI: 2.09, 7.51, *P* = 0.001), systolic blood pressure (SBP) (WMD: –2.96 mmHg; 95% CI: –4.11, –1.81, *P* < 0.001), and C-reactive protein (CRP) (WMD: –0.40, mg/l, 95% CI: –0.67, –0.14, *P* = 0.002) levels. In celiac patients as well as with an intervention duration of more than 48 weeks, GFD increased TC and HDL compared to non-celiac patients and with an intervention duration lower than 48 weeks, respectively. The results of the present study showed that GFD can have a significant and beneficial effect on HDL, SBP, and CRP.

## Introduction

Cardiovascular (CV) diseases, the main type of non-communicable diseases, were responsible for about 17.8 million deaths in 2017, and it is predicted to increase the number of deaths to 23.6 million by 2030.^(1,2)^ Apart from CVD mortality, imposing the heavy costs of the disease on individuals and society as well as reducing the quality of life are two serious challenges not only at the individual levels, but also at health system and macroeconomic levels.^(1,3)^ Therefore, due to the important and widespread role of CVD as a major health problem, it is necessary to identify and follow appropriate guidelines for the prevention and treatment of this disease. Glycaemic, insulin, and lipid disorders as metabolic risk factors, play a vital role in the onset and development of CVD.^(4–7)^ In addition to genetic factors, several environmental factors including, smoking, sedentary lifestyle and imbalanced dietary intake are important factors contributing to increase CVD risk factors.^(8,9)^ Therefore, interventions targeting modification of this risk factors such as hyperglycaemia, impaired insulin secretion, hyperlipidaemia and hypertension, are of significant importance in prevention and treatment of CVD.^(10)^

In this context, modifying the diet and using some special diets or micronutrients has attracted special attention.^(11)^ In fact, studies show that special diets, in addition to having fewer side effects than chemical drugs, are better predictors of various CVD risk factors and all causes related to its death.^(12)^ So that the reports of meta-analysis articles show the beneficial effects of several special diets including DASH, Palaeolithic diet, Mediterranean diet, and healthy Nordic diet on these risk factors.^(13–15)^ Another one of these diets that has recently attracted the attention of researchers and people is the gluten-free diet (GFD). According to the evidence, the number of people using GFD is increasing every day, and it seems that the value of the global industry of this diet has reached more than 6 billion dollars.^(16)^ Wheat, barley, and oats are rich sources of the complex protein known as gluten, which is made up of glutenin and prolamin.^(16)^ Recent research demonstrates the potential positive effects of GFD in a number of illnesses, including type I diabetes, obesity, and insulin resistance. Although GFD is acknowledged as the primary and prospective therapy for celiac disease (CD).^(17–19)^ Through the decrease of peripheral adipose tissue and fat cell size, GFD appears to have the potential to have positive impacts on CVD risk factors such insulin resistance, lipid profile, inflammatory variables, and blood pressure.^(17)^ Although, the role of GFD on blood glucose and lipid level have been investigated in several human studies, the finding are equivocal. In the several studies, people without CD had a significant reduction in insulin resistance factors and higher levels of HDL-C after receiving a GFD compared to people who consumed a normal diet.^(20–22)^ However, another study was observed no significant effects on CVD risk factors.^(20,23)^ Therefore, given these contradictory results, this study was conducted to estimate a more precise effect of GFD on CVD risk factors.

## Methods

### Search strategy

The Preferred Reporting Items for Systematic Review and Meta-analysis (PRISMA) criteria were followed for conducting this study.^(24)^ The study protocol has been previously registered with the PROSPERO database (registration number CRD42022365144). Without regard to language or time restrictions, a thorough search was carried out in the PubMed/MEDLINE, Web of Science, SCOPUS, and Embase databases from the beginning until July 12, 2022. The following medical subject were chosen to search the online databases: (“diet, gluten free ” OR “Gluten-Free Diet”) AND (“Glycated Hemoglobin A” OR HbA1c OR “Insulin Resistance” OR Insulin OR Glucose OR “Glucose Intolerance” OR Triglycerides OR Cholesterol OR “Cholesterol, HDL” OR “Cholesterol, LDL” OR “High-density lipoprotein” OR “Low-density lipoprotein” OR “Blood Pressure” OR “Arterial Pressure” OR “Hypertension” OR SBP OR DBP OR “C-reactive protein” OR CRP). To locate potentially overlooked qualifying trials, the reference lists of the papers that were collected and associated review articles were also manually searched.

### Eligibility criteria

Using titles, abstracts, or the complete texts of the studies, the authors separately removed duplicate articles, found and reviewed related articles. Following criteria were used to extract the articles, which was the final step: (1) articles with follow-up studies of one week or more, including prospective or retrospective single arm, (2) performed on paediatric and adult participants who underwent GFD (Individuals with and without CD), (3) reported the primary and secondary outcomes (HbA1c, fasting glucose sugar (FBS), insulin, Homeostatic Model Assessment for Insulin Resistance (HOMA-IR), total cholesterol (TC), triglyceride (TG), High-density lipoprotein-cholesterol (HDL-C), low-density lipoprotein-cholesterol (LDL-C), systolic blood pressure (SBP), diastolic blood pressure (DBP), and C-reactive protein (CRP)) at the baseline and after GFD. When a research reported the amount of a factor over several follow-up times, the longest or most recent follow-up period was taken into account. Exclusion criteria for our investigation included papers with duplicate or unclear data, systematic publications, other observational studies, studies with less than one week of follow-up, and studies with no response after contacting relevant authors.

### Data extraction and quality assessment

Information required for the article included mean and standard deviation (SD) HbA1c, FBS, insulin, HOMA-IR, TC, TG, HDL-C, LDL-C, SBP, DBP, and CRP at the baseline and after GFD, the name of the authors, year of publication, country, number of participants, percentage of male participants, type of population, mean age (year), studies design, and follow-up duration of the intervention. Two independent researchers reviewed relevant articles before extracting this data. Additionally, using the Newcastle-Ottawa Quality Assessment Scale, two writers independently evaluated the included publications’ quality.^(25)^

### Data synthesis and statistical analysis

The statistical analysis was done using RevMan 5.3 (The Nordic Cochrane Centre, Copenhagen, Denmark) software and STATA version 12.0 (Stata Corp, College Station, TX, USA). The mean and SD of variables at the baseline and after GFD were taken from each research to determine the weight mean differences (WMD), and the WMD was then calculated for each article using a random-effects models. Standard calculations were carried out to determine the mean and SDs when data were provided in a different manner.^(26,27)^ Furthermore, for studies that only reported standard error of the mean (SEM), SDs were obtained using the following formula: SD = SEM × √ n, where ‘*n’* is the number of subjects. *Q* Statistics and *I*-squared (*I* ^*2*^) were used to evaluate the heterogeneity status between studies. Insignificant, low, moderate, and high heterogeneity were identified with an *I* ^*2*^ values of 0–25%, 26–50%, 5–75%, and 76–100%, respectively.^(28)^ Pre-defined subgroup analysis based on participant type (celiac/non-celiac) and duration study follow-up was carried out to find possible causes of heterogeneity. To determine the contribution of each research to the total mean difference, a sensitivity analysis was used. Additionally, we used the Begg tests to assess the publication bias. To determine if publication bias influenced the stability of the total estimate when there was severe publication bias, we employed the trim and fill approach.^(29)^

## Results

**Fig. 1.**
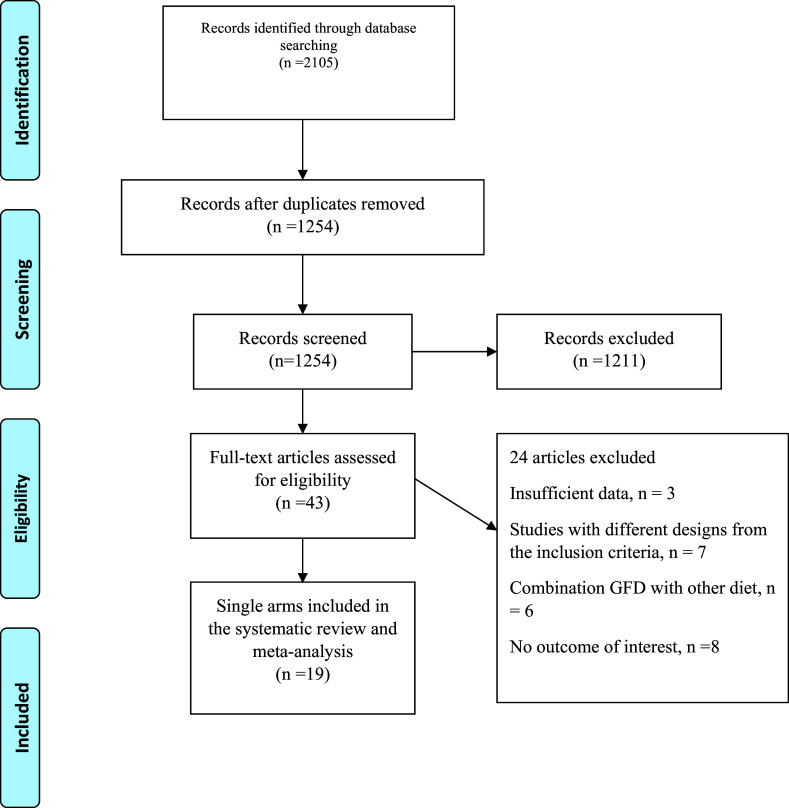
Flow chart of the included studies, including identification, screening, eligibility and the final sample included.

Process of selection is shown in Fig. 1. After searching the systematic databases, 2105 results were selected (one additional articles identified through other sources), with 1254 articles remaining after the elimination of duplicate studies. Then, after reviewing the abstract or title, 1211 studies did not meet the inclusion criteria. After retrieving the full text of the remaining 24 articles, nine articles were deleted due to insufficient data (absence of investigated variables or non-reporting of mean or standard deviation data and other transformable data) (*n* = 3), study design differing from the inclusion criteria (*n* = 7), combination GFD with other diet (*n* = 6), and absence of outcome of interest (*n* = 8). Finally, 19^(21,22,30–45)^ studies with 21 treatment arms met the eligibility and were included in our analysis.

## Study characteristics

Table 1 reveals the characteristics of the pooled articles. According to our studies, two studies have been conducted in the Americas, two studies in Asia and the rest in Europe. All studies were published between the years 1996–2021. In addition, according to our information, all studies were performed on both sexes and the percentage of males in the studies varied from 0% to 56%. The follow-up intervention of the studies was between 4 and 114 weeks. The mean age at the baseline varied between 7.6 and 58.5 years. In addition, according to the findings, 8 studies have been conducted as a prospective cohort, and the rest as a retrospective cohort. On the other hand, 4 studies on patients with type 1 diabetes mellitus (T1DM) and CD at the same time, 10 studies on patients with CD alone, 2 articles on T1DM, and finally 3 studies on people with rheumatoid arthritis, metabolic syndrome, and healthy individual have been done. Five studies for HbA1c and insulin, 7 articles for glucose, 3 studies for HOMA-IR, as well as, 11 articles for TC, TG and HDL, and 9 study with 11 arms for LDL provided data on the comparison of mean changes. Four studies reported the effect of GFD on SBP, DBP, and CRP level. In addition, the quality results of the articles are shown in Table 2.

**Table 1.** Characteristics of eligible studies

Number	Author (y)	Years	Country	Study design	Population	Mean age year	Male (%)	Sample size	Duration of study (wk)	Outcomes
1	Kaur *et al.*	2020	India	PC	T1DM and CD	25.7	56.0	15	48	HbA1c.
2	Bakker *et al.*	2012	Netherlands	RC	T1DM and CD	47	42.1	31	N/R	HbA1c.
3	Kaukinen *et al.*	1999	Finland	RC	T1DM and CD	N/R	N/R	22	48	HbA1c.
4	Neuman *et al.*	2020	Prague	PC	T1DM	10.2	50	20	48	HbA1c.
5	Marchi *et al.*	2013	Italy	RC	CD	N/R	45	20	28	TC, LDL, HDL, TG, SBP, DBP, CRP.
6	Zanini *et al.*	2013	Italy	RC	CD	35	29/5	547	144	FBS, Insulin, HOMA-IR, TC, LDL, HDL, TG.
7	Riezzo *et al.*	2014	Italy	PC	CD	34	25	20	48	TC, LDL, HDL, TG, SBP, DBP, CRP.
8	Salardi *et al.*	2017	Italy	RC	T1DM and CD	7/6	N/R	129	48	HbA1c, TC, LDL, HDL, TG.
9	Remes-Troche *et al.*	2019	Mexico	PC	CD/NCD	30	10	22	24	FBS, TC, LDL, HDL, TG.
10	Ehteshami *et al.*	2018	Iran	RC	Metabolic syndrome	58/55	26	23	8	FBS, Insulin, HOMA-IR, TC, LDL, HDL, TG, SBP,DBP.
11	Brar *et al.*	2006	USA	RC	CD	44/4	34	132	82	TC, LDL, HDL, TG.
12	Ciacci *et al.*	2002	Italy	RC	CD	27/9	23/3	390	7	TC.
13	Elkan *et al.*	2008	Sweden	RC	Rheumatoid arthritis	50	10/3	22	48	TC, LDL, HDL, TG, CRP.
14	Lewis *et al.*	2009	UK	PC	CD	51	N/R	100	48	FBS, TC, HDL, CRP.
15	Rea *et al.*	1996	Italy	PC	CD	N/R	N/R	23	48	TC, TG.
16	Pastore *et al.*	2003	Italy	RC	T1DM	16	N/R	14	24	FBS, Insulin, HOMA-IR.
17	Goddard *et al.*	2021	UK	PC	Health	29	9	11	4	FBS, Insulin, SBP, DBP.
18	Tortora *et al.*	2014	Italy	PC	CD	N/R	N/R	254	48	FBS, HDL.
19	Capristo *et al.*	2005	Italy	RC	CD	31.4	0	18	104	Insulin.

N/R, not reported; PC, prospective cohort; RC, retrospective cohort; T1DM, Type 1 diabetes mellitus; CD, celiac diseases; NCD, none celiac diseases; TC, total cholesterol; LDL, low-density lipoprotein; HDL, high-density lipoprotein; TG, triglyceride; FBS, fasting blood sugar; HOMA-IR, Homeostatic Model Assessment for Insulin Resistance; SBP, systolic blood pressure; DBP, diastolic blood pressure; CRP, C-reactive protein.

**Table 2.** Risk of bias assessment according to the Newcastle-Ottawa Quality Assessment Scale tool

Study	Selection	Comparability	Outcome/exposure
Kaur *et al.*	***	*	***
Bakker*et al.*	****	**	**
Kaukinen *et al.*	**	**	**
Neuman *et al.*	***	–	***
Marchi *et al.*	**	**	***
Zanini *et al.*	****	*	**
Riezzo *et al.*	**	**	***
Salardi *et al.*	**	**	***
Remes-Troche *et al.*	****	–	**
Ehteshami *et al.*	***	*	***
Brar *et al.*	**	***	***
Ciacci *et al.*			
Elkan *et al.*	**	***	***
Lewis *et al.*	**	***	***
Francesco *et al.*	**	**	***
Pastore *et al.*	**	**	***
Goddard *et al.*	****	–	**
Tortora *et al.*	***	*	***
Capristo *et al.*	**	***	***

Quality assessment of articles were classified into low (< 5 stars), moderate (5–7 stars) and high quality (> 7 stars).

### Meta-analysis

#### The effect of GFD on HbA1c, fasting glucose, insulin, and HOMA-IR

**Fig. 2.**
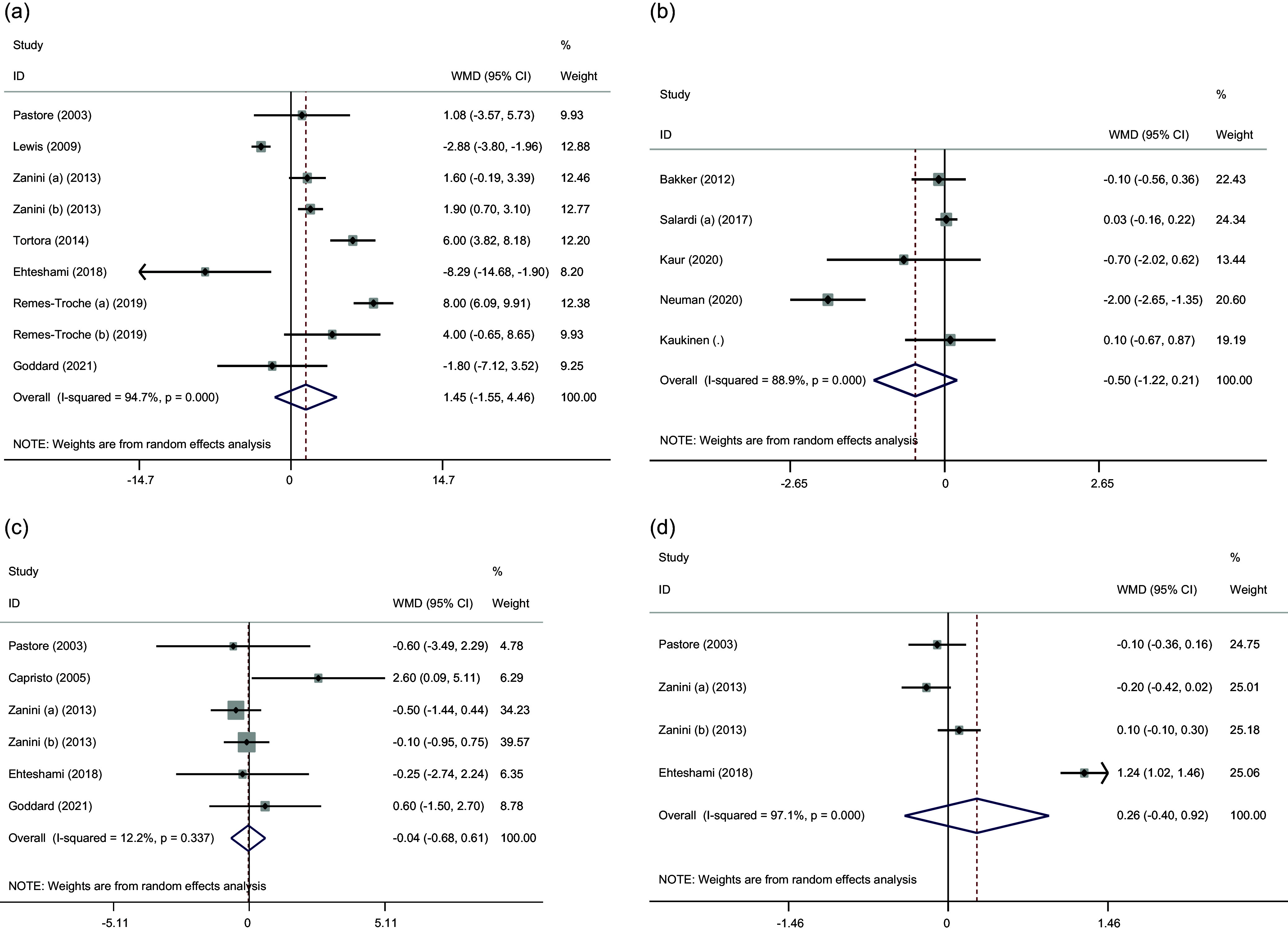
Forest plots from the meta-analysis of investigating the effects of gluten-free diet on (a) HbA1c, (b) glucose, (c) insulin, and (d) HOMA-IR. WMD: weighted mean.

Pooled results from the random-effects model indicated that the GFD has no significant effect on any of the factors of glucose metabolism, including fasting glucose (WMD: –0.50 mg/dl, 95% CI: –1.22, 0.21, *P* = 0.343), insulin (WMD: –0.04 µU/ml, 95% CI: –0.68, 0.61, *P* = 0.906), HbA1c (WMD: 1.45, 95% CI: –1.55, 4.46, *P* = 0.169), and HOMA-IR (WMD: 0.26, 95% CI: –0.40, 0.92, P= 0.437). Furthermore, significant heterogeneity was noted for HbA1c (Cochran *Q* test, *P* < 0.001, *I* ^2^ = 94.7%), HOMA-IR (Cochran *Q* test, *P* < 0.001, *I* ^2^ = 97.1%), and fasting glucose (Cochran *Q* test, *P* < 0.001, *I* ^2^ = 88.9%) among the studies. However, there was no evidence of significant between-study heterogeneity for insulin (Cochran *Q* test, *P* = 0.337, *I* ^2^ = 12.2%) (Fig. 2). Also, subgroup analyses for these outcomes did not show significant results (Supplementary Fig. 1–3).

#### The effect of GFD on lipid profile

**Fig. 3.**
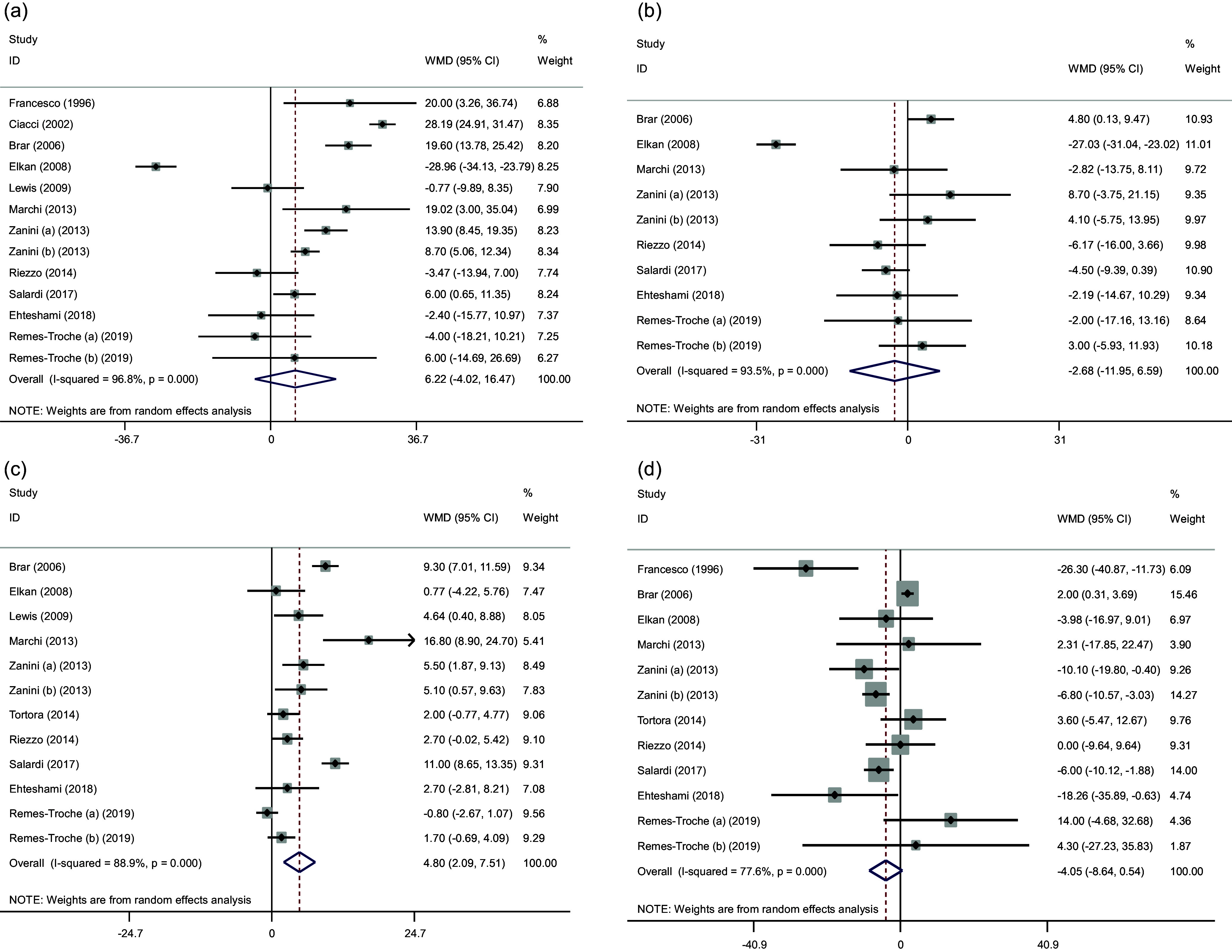
Forest plots from the meta-analysis of investigating the effects of gluten-free diet on (a) cholesterol, (b) LDL, (c) HDL and (d) TG. WMD: weighted mean.

Pooled data indicated a significant efficacy in increasing serum HDL (WMD: 4.80 mg/dl, 95% CI: 2.09, 7.51, *P* = 0.001) following adherence to a GFD. However, no significant beneficial effects on TC (WMD: 6.22 mg/dl, 95% CI: –4.02, 16.47, *P* = 0.232), LDL-C (WMD: –2.68 mg/dl, 95% CI: –11.95, 6.59, *P* = 0.571), and TG levels (WMD: –4.05 mg/dl, 95% CI: –8.64, 0.54, *P* = 0.084) concentration was reported after consumption of GFD. Significant heterogeneity was observed between these articles for TC (Cochran *Q* test, *P* < 0.001, *I* ^2^ = 96. 8%), TG (Cochran *Q* test, *P* < 0.001, *I* ^2^ = 77.6%), LDL-C (Cochran *Q* test, *P* < 0.001, *I* ^2^ = 93.5%), and HDL levels (Cochran *Q* test, *P* < 0.001, *I* ^2^ = 88.9%) (Fig. 3).

The subgroup results reported that following a GFD increased TC and HDL in celiac patients compared to non-celiac patients and with an intervention duration of more than 48 weeks. Also, the effect of this diet on the increase of LDL was greater during the intervention of more than 48 weeks (Supplementary Figs. 4–7).

#### The effect of GFD on SBP and DBP

**Fig. 4.**
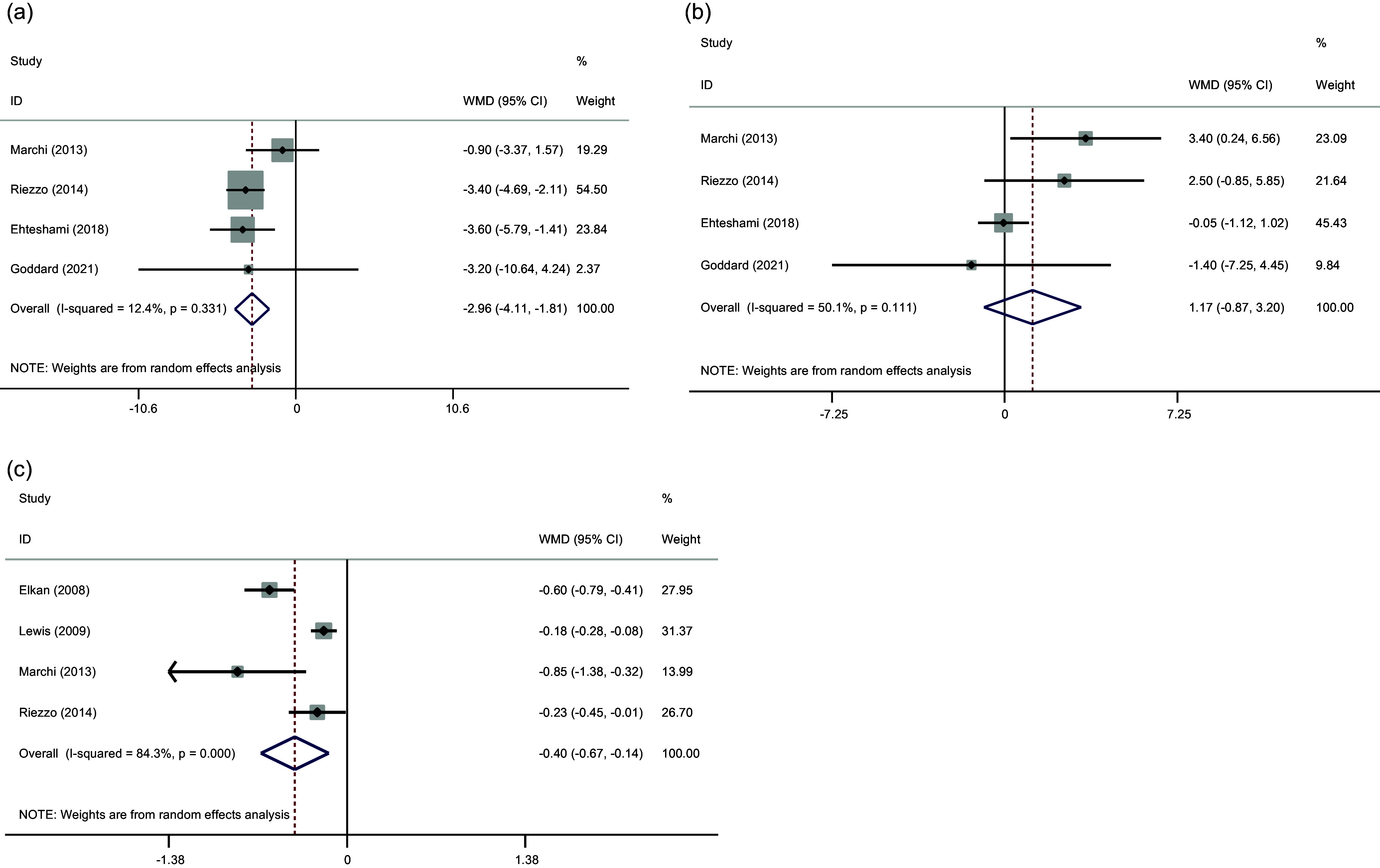
Forest plots from the meta-analysis of investigating the effects of gluten-free diet on (a) SBP, (b) DBP, (c) CRP.WMD: weighted mean.

The pooled effect sizes from three studies indicated that GFD had a significant effect on SBP (WMD: –2.96 mmHg; 95% CI: –4.11, –1.81, *P* < 0.001). But no significant effect was observed on DBP (WMD: 1.17 mmHg; 95% CI: –0.87, 3.20, *P* = 0.262). However, for SBP and DBP, the detected heterogeneity was not significantly high (Cochran *Q* test, *P* = 0.331, *I* ^2^ = 12.4% for SBP and Cochran *Q* test, *P* = 0.111, *I* ^2^ = 50.1% for DBP), respectively (Fig. 4).

#### Effect of GFD on CRP

Three studies reported data for serum as outcome measures. The results from our meta-analysis indicated a significant reduction of CRP (WMD: –0.40, mg/l, 95% CI: –0.67, –0.14, *P* = 0.002) levels following GFD consumption. In addition, significant heterogeneity was noted among the analysed studies for CRP (Cochran *Q* test, *P* < 0.001, *I* ^2^ = 84.3%) (Fig. 4).

### Sensitivity analysis

In order to discover the effect of each article on the pooled effect size for TC, LDL-C and HDL-C, TG, HbA1c, glucose, insulin, CRP, SBP, and DBP levels, we step by step discarded each trial from the analysis. The leave-one-out sensitivity analysis indicated the robustness of the results (Supplementary Figs. 8–10).

### Publication bias

Evaluation of publication bias by visual inspection of funnel plot and Egger’s test demonstrated no evidence for publication bias in the meta-analysis of GFD on CRP (*P* = 0.497), SBP (*P* = 0.174), DBP (*P* = 1.00), TC (*P* = 0.903), LDL-C (*P* = 0.655) and HDL-C (*P* = 0.493), TG (*P* = 0.681), glucose (*P* = 0.297), insulin (*P* = 0.851), HOMA-IR (*P* = 0.497), and HbA1C (*P* = 0.327) levels (Supplementary Figs. 11–13).

## Discussion

The findings of this meta-analysis indicated that GFD has no significant effect on HbA1c, fasting glucose, insulin, and HOMA-IR. Thus, these findings do not support the recommendation to exclusion of the gluten of the feeding in clinical practice to the improvement of parameters of glucose metabolism.

On the other hand, regarding lipid profile, results of the meta-analysis showed significant effect of the GFD in increasing serum HDL. In celiac patients as well as with an intervention duration of more than 48 weeks, GFD increased TC and HDL compared to non-celiac patients and with an intervention duration lower than 48 weeks, respectively. The effect of this diet on the increase of LDL also was greater with a higher duration of the intervention.

Regarding HDL cholesterol, it is proposed that the elevated blood HDL levels following GFD are reasonably attributed to mucosal healing and, as a result, to an improvement in intestinal absorption of HDL and apo-A1), the primary apo-protein of circulating HDL cholesterol.^(23,46)^ Additionally, given that Apo-A1 secretion and HDL cholesterol synthesis are decreased in the small intestinal mucosa of CD patients who are not receiving treatment, the significant increase in serum HDL levels seen in celiac patients with the longer intervention duration compared to non-celiac patients with the shorter intervention duration is also plausible.^(46)^

As for the increase in serum levels of total and LDL cholesterol after intervention with a GFD, this may be a result of the concomitant increase in serum HDL levels as an effect of the GFD.^(22)^ It is noted that the serum HDL level has greater relevance as a cardiovascular risk factor than total cholesterol and is independent of LDL levels, despite evidence that a GFD also increases serum levels of total and LDL cholesterol, which may increase the risk of cardiovascular disease.^(47,48)^ This might not be true necessary given that genetic studies and clinical trials generally show that the relationship between HDL-C and CVD is not causal.^(49)^

Concerning blood pressure, results of the meta-analysis showed that GFD had a significant effect only on SBP. The possible mechanisms to explain the reduction in blood pressure following GFD are still unknown. The possible mechanisms to explain the reduction in blood pressure after the GFD are still unknown. Contrarily, it has been shown that the angiotensin I-converting enzyme (ACE) is inhibited by gliadin (a protein found in gluten). Because ACE destroys bradykinin, a vasodilator, and transforms angiotensin I into angiotensin II, a vasoconstrictor, its suppression may help lower blood pressure.^(50)^ Therefore, additional research is required to learn more about the mechanisms that may underlie the benefits of a GFD on blood pressure.

Regarding CRP, results of the meta-analysis showed a significant reduction of CRP levels following GFD. This finding suggests possible decreased inflammatory response secondary to gluten abstinence indicated by a significant decrease in inflammatory markers such CRP levels serum.^(51)^

In a systematic study in 2018 by Potter et al to investigate the effect of this diet on cardiovascular risk factors, the results showed that GFD increases TC, HDL-C, FBS, and BMI. However, no significant effect was reported on LDL-C, TG, and BP. In general, most of its findings were different from our findings. However, this study differed in design from ours in several ways. In Potter’s study, unlike our study, the study of the effect of this diet was only limited to patients with CD and only systematically reviewed the results without conducting a meta-analysis, and also the overall quality of the study was reported to be low, which ultimately all these factors can affect the final results and cause contradictory results between two studies.

Our study has a number of advantages. The current study is the first meta-analysis to look into how GFD might affect patients with and without CD in terms of their cardiometabolic risk factors. We made an effort to incorporate every study that would have met the inclusion criteria in our meta-analysis so that the large number of studies would boost the reliability of the findings. In addition, we conducted a subgroup analysis to identify the causes of high heterogeneity in study results. The subgroups were divided according to the population type and the length of follow-up.

The inclusion of studies without an appropriate control group, different inclusion criteria for study participants, the absence of other common treatments, prior medical history, various and variable levels of disease activity, and ultimately different essential characteristics, such as age, sex, BMI, duration of CD and other diseases that these may contribute to population heterogeneity and eventually have an impact on the outcomes, are all limitations of the current study.

In summary, the results of this meta-analysis showed that the GFD showed a beneficial impact on some cardiometabolic risk factors such as an increase in serum HDL cholesterol levels, a reduction in SBP and serum CRP levels. However, no significant effect was observed on parameters of glucose metabolism. It is important to take into account also the differences in the magnitudes of the effects of the GFD when comparing their effect in celiac and non-celiac patients, as well as the duration of the intervention period.

A GFD does not automatically mean a better diet. If care is made to choose whole-grain goods, include more vegetables, and choose items with reduced energy density, a GFD may be a well-balanced diet.^(52)^ Therefore, other dietary strategies to reduce cardiometabolic risk factors may be taken into consideration in the absence of gluten-related illnesses.

## Supporting information

Rohani et al. supplementary material 1 Rohani et al. supplementary material

Rohani et al. supplementary material 2 Rohani et al. supplementary material

Rohani et al. supplementary material 3 Rohani et al. supplementary material

## Data Availability

Data available on request due to privacy/ethical restrictions.

## Abbreviations

**GFD:** Gluten-free diet; **CV:** Cardiovascular; **HDL:** High-density lipoprotein; **SBP:** Systolic blood pressure; **CRP:** C-reactive protein; **CD:** Celiac disease; **FBS:** Fasting glucose sugar; **HOMA-IR:** Homeostatic model assessment for insulin resistance (HOMA-IR); **TC:** Total cholesterol; **TG:** Triglyceride; **LDL:** Low-density lipoprotein; **DBP:** Diastolic blood pressure

## Supplementary material

The supplementary material for this article can be found at https://doi.org/10.1017/jns.2024.39
