# Supplementary material for: Impact of gluten-free diet (GFD) on some of cardiovascular risk factors: a systematic review and meta-analysis
Source: J Nutr Sci. 2024 Sep 18;13:e37. doi: 10.1017/jns.2024.39 (PMC11428062; doi:10.1017/jns.2024.39)
Supplement: Rohani et al. supplementary material 1 — Rohani et al. supplementary material [file S2048679024000399sup001.docx]

| a)   | b)   |
| --- | --- |
|  |  |
| Supplemental Figures 1. Forest plots from the meta‐analysis of clinical trials investigating the effects of GFD supplement on fasting glucose on a) duration (week) and b) type of participants (celiac/none-celiac). WMD: weighted mean | |

| a)   | b)   |
| --- | --- |
|  |  |
| Supplemental Figures 2. Forest plots from the meta‐analysis of clinical trials investigating the effects of GFD supplement on insulin on a) duration (week) and b) type of participants (celiac/none-celiac). WMD: weighted mean | |

| a)   | b)   |
| --- | --- |
|  |  |
| Supplemental Figures 3. Forest plots from the meta‐analysis of clinical trials investigating the effects of GFD supplement on HOMA-IR on a) duration (week) and b) type of participants (celiac/none-celiac). WMD: weighted mean | |

| a)   | b)   |
| --- | --- |
|  |  |
| Supplemental Figures 4. Forest plots from the meta‐analysis of clinical trials investigating the effects of GFD supplement on TC on a) duration (week) and b) type of participants (celiac/none-celiac). WMD: weighted mean | |

| a)   | b)   |
| --- | --- |
| Supplemental Figures 5. Forest plots from the meta‐analysis of clinical trials investigating the effects of GFD on LDL-C on a) duration (week) and b) type of participants (celiac/none-celiac). WMD: weighted mean | |

| a)   | b)   |
| --- | --- |
|  |  |
| Supplemental Figures 6. Forest plots from the meta‐analysis of clinical trials investigating the effects of GFD on HDL-C on a) duration (week) and b) type of participants (celiac/none-celiac). WMD: weighted mean | |

| a)   | b)   |
| --- | --- |
| Supplemental Figures 7. Forest plots from the meta‐analysis of clinical trials investigating the effects of GFD on TG on a) duration (week) and b) type of participants (celiac/none-celiac). WMD: weighted mean | |
