# Supplementary material for: Impact of gluten-free diet (GFD) on some of cardiovascular risk factors: a systematic review and meta-analysis
Source: J Nutr Sci. 2024 Sep 18;13:e37. doi: 10.1017/jns.2024.39 (PMC11428062; doi:10.1017/jns.2024.39)
Supplement: Rohani et al. supplementary material 3 — Rohani et al. supplementary material [file S2048679024000399sup003.docx]

| a)   | b)   |
| --- | --- |
| c)   | d)   |
| Supplementary figures 8. Leave-one-out sensitivity analysis of the effects of GFD on (a) glucose, b) insulin, c) HbA1c b) HOMA-IR | |

| a)   | b)   |
| --- | --- |
| c)   | d)   |
| Supplementary figures 9. Leave-one-out sensitivity analysis of the effects of GFD on (a) cholesterol, (b)LDL, c) HDL and d) TG.. | |

| a)   | b)   |
| --- | --- |
| c)   |  |
| Supplementary figures 10. Leave-one-out sensitivity analysis of the effects of GFD on (a) SBP, (b)DBP, c) CRP. | |
